# Gender Differences in the Perception of Personalized Half-Nude Female Bodies

**DOI:** 10.3389/fpsyg.2017.01529

**Published:** 2017-09-08

**Authors:** Sarita Silveira, Katrin M. Elvers, Kai Fehse, Marco Paolini

**Affiliations:** ^1^Institute of Medical Psychology, Ludwig Maximilian University of Munich Munich, Germany; ^2^Clinic and Polyclinic for Radiology, Ludwig Maximilian University of Munich Munich, Germany

**Keywords:** body representations, framing, aesthetics, gender differences, functional magnetic resonance imaging

## Abstract

In the current study, we investigated how the perception of half-nude female body representations is altered by framing with information about the presented person. Images from tabloid newspapers were presented to male and female observers, and rated according to their aesthetic appeal while neurofunctional correlates were assessed using functional magnetic resonance imaging. While a generally stronger appetitive response might be expected in men, our results show a significant interaction between framing condition and gender of the observer. Men rated female bodies as more pleasing when presented without personal information, whereas women expressed more aesthetic appeal when information was added. Neuroimaging data revealed gender differences in processing body representations with additional personal information. In women, there was a stronger involvement of the anterior cingulate cortex and adjacent ventromedial prefrontal cortex, and in male observers a higher engagement of the bilateral inferior parietal cortex, when compared to each other respectively. These gender differences in framing effects particularly highlight higher aesthetic appeal and reward processing in women when female bodies are personalized.

## Introduction

For every person, specific objective information can be related to personal identity. In particular names are used to identify a person, thus in history of war, the replacement of a name by a number was used as a strategy of dehumanization. Media oftentimes presents us with depictions of female bodies, not necessarily for the explicit purpose to trigger sexuality and erotic appeal, but to attract people’s attention or motivate a certain attitude or behavior, however, instilling a discourse about women as objects. Thus media and marketing may use women to reach economic goals. The current study investigates gender differences in the perception of female bodies and their ‘de-objectification’ by using additional personal information.

In addressing the ways, in which information can alter the perception of a person, one might refer to the concept of top–down information processing, which is complementary to bottom-up processing of the physiological properties of a stimulus (Kinchla and Wolfe, 1979; Ullman, 1995; Engel et al., 2001). The notion of top–down modifications in the perception of physiologically identical stimuli is known as framing, a term stemming from linguistics, sociology and psychology (Goffman, 1974; Lakoff and Johnson, 1980; Tversky and Kahneman, 1985). Framing is as such based on a context, which determines visual perception according to implicit knowledge systems that can be understood as anthropological universals (Pöppel and Bao, 2011; Bao and Pöppel, 2012). Contextual information can modulate both cognitive and affective aspects in processing a stimulus. ‘Attribute framing’ describes those effects that result from the positive or negative valence of stimulus attributes on its evaluation (Levin et al., 1998). In this line, previous research shows that pairing odors with positive or negative words influences the perception of smell with regard to pleasantness (de Araujo et al., 2005). This positive as compared to negative attribute framing exhibited higher levels of neural activation in the orbitofrontal cortex (OFC), which is among the brain regions related to reward processing. Beside affective responses, conceptual and contextual framing engages higher cognitive functions based on prior expectations or knowledge systems, which has been linked to an involvement of the hippocampus, temporal, prefrontal, and parietal brain regions associated with memory, imagery, and attention (McClure et al., 2004; Silveira et al., 2015). Evidence is provided that those top–down cues, particularly in terms of additional information, can influence aesthetic visual processes (Cupchik et al., 1994; Russell, 2003; Leder et al., 2006; Kirk et al., 2009; Silveira et al., 2015): when presenting identical artworks in the context of different semantic frames like title, origin, or authenticity, perceptual processes are modulated, accompanied by complex brain activation patterns, particularly in the frontal and parietal lobes.

Photographs of nude or half nude bodies appeal to concepts of beauty and aesthetics, and can be understood as a subset of visual art in general (Seltzer, 2011). Female body representations have previously been studied as appetitive stimuli, which are directly related to sexual behavior. In these studies, several reward-related brain areas including the OFC, ventral striatum, and anterior cingulate cortex (ACC) were identified as neural correlates of erotic-related processes in men (Redouté et al., 2000, 2005). Besides, particularly parts of the ACC have been associated with an affective component in visually evoked sexual arousal in men (Stoleru et al., 1999). Also parietal brain areas seem to play a pivotal role when male observers watch erotic stimuli, which might indicate attentional processes (Mouras et al., 2003).

In order to investigate gender differences in the effects that framing with personal information has on the perception of body depictions, we presented male and female participants with half nude photographs of women. We chose to limit our study to the observation of female body representations due to their ecological validity, with the disproportionate focus of sexualized images of women versus men in the media. When it comes to differences between men and women in processing sexually arousing visual stimuli, assumptions about stronger appetitive responses in men were expanded by findings that the depicted sex of the stimulus plays a bigger role for men and the context for women (Rupp and Wallen, 2008). Neuroimaging research on the processing of visual sexual stimuli provides evidence for generally similar activation patterns in men and women, e.g., in the ACC, OFC and insula, yet higher involvement of particular brain areas like hypothalamus, thalamus, amygdala, or ventral striatum in men (Karama et al., 2002; Hamann et al., 2004). With regard to aesthetic processes, gender differences have previously been found in parietal brain regions (Cela-Conde et al., 2009). In general, we expected affective as well as cognitive framing effects in cortical brain areas to correspond to aesthetic appeal, appetitive motivation, and attention when additional personal information is given to images of half nude female bodies.

## Materials and Methods

### Participants

Seventeen right-handed German speakers (nine female; mean age 37, *SD* = 6.11 years) with normal or corrected-to-normal vision participated. We included male and female participants with all sexual orientations. They were recruited via an announce in a local tabloid newspaper. The study was conducted in accordance with the Declaration of Helsinki and approved by the ethics committee of the medical faculty of the Ludwig Maximilian University of Munich. Informed written consent was obtained from each participant and they received financial reward.

### Materials and Apparatus

In a behavioral pilot study, 100 female names and 100 places were evaluated by 300 participants (140 females, mean age 32.6 years, age range 21–46 years) according to their positive or negative value (“how much does this name/place appeal to you”) on a five-point Likert scale ranging from 1 = not at all at all to 5 = very much. Ratings were collected in an online survey. Names were chosen from tables of the most widely used female names in the past 5 years in Germany. The 100 biggest cities of the world were used as places. Those 18 names and cities with the highest mean scores were selected for the fMRI scanning session.

As stimuli we used 36 images that were split into two groups, leaving 18 images per experimental condition. They were taken from the database of a digital publishing house with permission of the administrators. The depicted female body representations were topless. All images were equalized in luminance and resized on a fixed image area of 250.000 pixels. A random selection of 18 images was distorted using a mosaic function (Photoshop CS3, Adobe Systems) and utilized as a control condition.

The study was conducted with a 3T whole body system (Achieva, Philips Healthcare, Best, The Netherlands) at the University Hospital LMU Munich. For blood-oxygen-level dependency (BOLD) imaging T2^∗^-weighted EPI sequence was used (TR = 2500 ms, TE = 30 ms, FA = 80°, 38 axial slices, slice thickness = 3 mm, no inter-slice gap, ascending acquisition, FOV = 448 mm × 448 mm, matrix = 64 × 64, in-plane resolution = 3 mm × 3 mm).

### Procedure

A block design was used consisting of eight blocks per experimental condition. Each block comprised three different images, displayed for 4000 ms respectively with an inter stimulus interval of 250 ms. Each block was followed by 6000 ms displaying a fixation cross. The order of stimuli and blocks was pseudo-randomized using a stimulus delivery software (Presentation 15.1, Neurobehavioral Systems, Berkeley, CA, United States). Participants viewed the presented images via a mirror attached to the MRI head-coil. They were asked to rate the depicted women as either attractive or unattractive by pressing the left or right button of an MRI compatible response device (LUMItouch, Photon Control, Inc., Burnaby, BC, Canada). In the two experimental conditions, the body representations were presented either without further information or with a name and place added. We created two versions of stimulus-condition-combinations, i.e., specific female bodies were presented to half of the participants in the framing and to the other half in the non-framing condition. Prior to the scanning session participants were told that the information provided is background information applicable to the corresponding woman.

### Data Processing and Analysis

Statistical analysis was calculated with MATLAB (MathWorks, Inc.) and the Statistical Package for the Social Sciences (SPSS Statistics 19.0; IBM). MRI data was analyzed using Statistical Parametric Mapping software (SPM8, Wellcome Department of Cognitive Neurology, London, United Kingdom^1^). Anatomical description was done referring to the Automatic Anatomic Labeling (AAL) (Tzourio-Mazoyer et al., 2002) atlas from the Wake Forest University (WFU) Pickatlas (Advanced NeuroScience Imaging Research Laboratory, Winston-Salem, NC, United States).

The first five volumes of the functional scans were discarded due to possible instabilities of the magnetic field. In preprocessing the data, images were 3D motion corrected using a six parameter rigid body spatial transformation, realigned, and spatially normalized to the EPI template (Evans et al., 1993). For spatial smoothing to minimize noise and residual differences between participants, images were convolved with an isotropic Gaussian kernel of 8 mm full width at half maximum (FWHM).

In a first step of statistical analyses, both experimental conditions were modeled with onsets and durations of blocks for each condition by a boxcar function convolved with a hemodynamic response function. T-contrasts were then calculated on a first level of statistical analyses for the data of every participant respectively, using a contrast weight of 1 for each experimental condition and a contrast weight of -1 for the mosaic control condition.

In a second step of analyses, those individual contrast images were used in a full factorial model with the two experimental conditions representing two within-subject factors and participant number as well as gender as between-subject factors. We calculated bi-directional contrasts comparing the two experimental conditions, i.e., when observing images with versus without additional information, and comparing male and female participants for each experimental condition respectively. Contrasts were calculated to compare factor levels using a significance level of *p* < 0.001.

In analyzing the behavioral data, we calculated a multivariate analysis of variance with reaction time and aesthetic appreciation as dependent variables, experimental condition and gender of the participant as fixed factors. We also included the image of a specific female body representation as well as participant number as nominal covariates to control for effects of specific characteristics of participants or stimuli. Significance levels were set *p* < 0.05.

## Results

### Behavioral Data

Regarding covariates, we found that individual differences between participants significantly influence both reaction times, *F* = 10.72, *p* = 0.001, η^2^ = 0.012, and positive or negative responses toward the presented images, *F* = 9.78, *p* = 0.002, η^2^ = 0.011. There was a significant effect of gender on both aesthetic appreciation, *F* = 17.51, *p* < 0.001, η^2^ = 0.020, and reaction times, *F* = 19.79, *p* < 0.001, η^2^ = 0.023. A significant framing effect of additional information was found only for reaction times, *F* = 12.23, *p* < 0.001, η^2^ = 0.014. However, we found a marginally significant interaction between gender of participant and experimental condition on aesthetic appreciation, *F* = 3.35, *p* = 0.068, η^2^ = 0.004. This interaction showed more positive responses of women and less positive responses of men toward half nude female body representations when additional information was given (**Table 1**).

**Table 1:** Mean aesthetic appreciation and reaction times (and standard deviations) of men and women while observing half nude female body representations.

	Men (*N* = 7)		Women (*N* = 9)	
	No	Additional	No	Additional
	information	information	information	information
Positive response in %	59 (0.49)	55 (0.50)	39 (0.49)	49 (0.50)
Reaction time in ms	1553 (599)	1762 (655)	1364 (658)	1482 (629)


### Neuroimaging Data

#### Framing Effect

**FIGURE 1:**
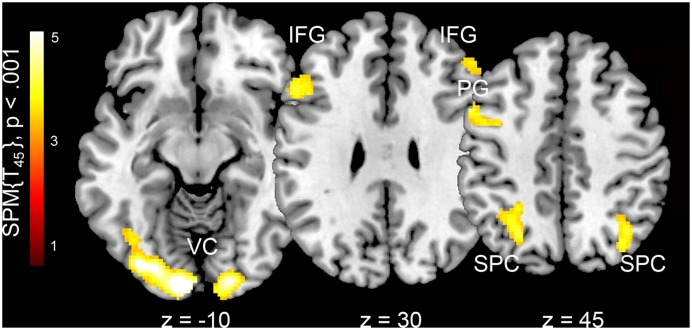
Neural correlates of framing female body representations with additional personal information in both male and female observers. VC, visual cortex; IFG, inferior frontal gyrus; PG, precentral gyrus; SPC, superior parietal cortex. Z-coordinates are in the MNI stereotactic space.

We found significant differences in BOLD activations when female body photographs were presented with compared to without additional information. When names and places of living were added, images were processed with higher levels of activation in the visual cortex (calcarine gyrus, lingual gyrus, fusiform gyrus, middle and inferior occipital cortex), bilateral superior parietal cortex, and bilateral inferior frontal cortex (**Table 2** and **Figure 1**). The reverse contrast revealed stronger involvement of the bilateral superior temporal gyrus and left insula when pictures were presented without personal information.

**Table 2:** Neural correlates of viewing female bodies with and without personal information.

	Coordinates				
Brain region	*x*	*y*	*z*	Size in voxels	*z*-Statistics
**Personal information > No information**					
Visual cortex				2853	
L calcarine gyrus	-12	-94	-10		5.88
L inferior occipital gyrus	-34	-82	-10		4.91
R lingual gyrus	14	-98	-6		4.55
Parietal cortex				1710	
L superior parietal cortex	-24	-74	58		4.98
R superior parietal cortex	34	-70	56		4.79
L inferior parietal cortex	-26	-60	38		4.37
L precentral gyrus	-46	4	52	659	4.01
L inferior frontal gyrus	-52	22	28		3.87
R inferior frontal gyrus	42	30	22	139	3.33
**No information > Personal information**					
L insular gyrus	-34	-16	16	317	4.79
L superior temporal gyrus	-54	-10	6	201	4.73
R superior temporal gyrus	64	-28	10	565	3.98


*L, left; R, right; the x, y, and z coordinates are in the MNI stereotactic space. Z-scores from t-tests, significance level p < 0.001.*

#### Gender Effect

When contrasting how depictions of half nude female bodies are processed in men and women without additional information, there was no gender difference. However, when names and places of living were given, men and women processed the female body depictions differently. In women, we found a stronger involvement of the ACC and adjacent ventromedial prefrontal cortex (vmPFC) as well as in the bilateral lingual gyrus and right fusiform gyrus when compared to male participants. The reverse contrast revealed higher BOLD activations in the bilateral inferior parietal cortex (**Table 3**).

**Table 3:** Gender differences in viewing female body representations that are framed with additional personal information.

	Coordinates				
Brain region	*x*	*y*	*z*	Size in voxels	*z*-Statistics
**Men > Women**					
R inferior parietal cortex	60	-44	46	242	4.32
L inferior parietal cortex	-46	-56	58	385	4.04
**Women > Men**					
Ventromedial prefrontal cortex	6	28	-8	852	5.18
Anterior cingulate cortex	-6	32	0		4.29
Visual cortex				364	
R lingual gyrus	8	-68	0		4.90
L lingual gyrus	-16	-74	2		3.89
R fusiform gyrus	42	-28	-24	198	4.55


*L, left; R, right; the x, y, and z coordinates are in the MNI stereotactic space. Z-scores from t-tests, significance level p < 0.001.*

## Discussion

In the current study, we investigated the effects of personal information on the perception of half nude female body representations in male and female observers. A general framing effect was found to involve brain areas in the bilateral superior parietal cortex, and inferior frontal lobe. These regions can be associated with semantic decoding and word comprehension in the visual modality (Booth et al., 2002; Turkeltaub et al., 2003). Thus, results derived from this contrast point to linguistic processes when semantic framing cues are given. Images with additional information were also accompanied by higher activation levels in the visual cortex, which might be explained by the additional visual input in the framing condition. A scrambled word control condition might be considered in future replications of this study to control for whether the stronger incurrence of the visual cortex is a by product of more visual information or related to framing effects on pictorial information processing. With regard to aesthetic processes, superior parietal regions have previously been associated with positive aesthetic judgments in general (Cela-Conde et al., 2009; Cupchik et al., 2009) and the observation of aesthetic body representations in specific (Lutz et al., 2013), thus in case of our study, their involvement might correspond to the positively valenced framing attributes.

In defining depictions of half nude bodies as aesthetic stimuli, responses to those photographs can be embedded in theories on emotions in aesthetic processes. Referring to James (1884) theory on emotions, aesthetic affective responses can be understood as embodied by nature. Thus in a reactive mode of perception, specific stimuli are linked with bodily sensations corresponding to aesthetic pleasure and arousal (Cupchik, 1995). This type of response might be reflected in an involvement of the left posterior insular cortex when observing female body representations without additional information. The insula has repeatedly been associated with diverse emotional qualities, and is supposed to play a crucial role in bridging bodily and affective states (Craig, 2002). This might indicate a stronger body-directed nature of responses, likely to be associated with sexual arousal (Karama et al., 2002) as well as with aesthetic pleasure (Cupchik et al., 2009) when no personal information was added to a body representation. Differential involvement of the bilateral superior temporal gyrus might be explained by the effect that additional information has on the evaluation of aesthetic appeal. A previous study shows that task dependent attention to facial expressions goes along with higher activation levels in the temporal lobe as compared to attention to facial gender (Critchley et al., 2000). Thus, additional information about the presented women might shift the focus away from the aesthetic appeal itself. Indices of personal information can thus be used for attribute framing, affecting visual, attentional, and top–down processing of body representations.

On a behavioral level, we found overall between-subject differences in explicit evaluations of aesthetic appeal. Men were more likely to rate the images as attractive than women. Additional information was generally associated with longer response times, yet, an effect of framing on the value of aesthetic appreciation was different for male and female observers. In men, responses were more positive toward half nude female body representations when no additional information about the person was given, whereas women showed more positive ratings when the female body was presented with a name and place of living.

This result is corroborated by the finding that the perception of female body representations with added information corresponds to higher activation levels in the ACC and adjacent vmPFC in women as compared to men. These paralimbic areas have previously been associated with the processing of erotic stimuli (Redouté et al., 2000, 2005). It was suggested that their engagement is linked to an affective component of erotic processing (Stoleru et al., 1999). In male compared to female observers, on the other hand, we found a higher involvement of the bilateral inferior parietal cortex. In visual perception, the inferior parietal cortices are parts of the dorsal stream, associated with visuo-spatial processes including spatial attention-shifts (Corbetta et al., 1995; Yantis et al., 2002). Our result also corresponds to previous findings that linked parietal brain regions to attentional processes in men when they viewed sexual visual stimuli (Mouras et al., 2003). In this regard, processing visual features of female body representations is accompanied with altered spatial attention in men, possibly indicating even stronger voluntary attentional control (Hopfinger et al., 2000).

With the limitation of stimulus material to depictions of female bodies, these results cannot be generalized to sexual visual stimuli *per se*. Assuming that the sexual orientation of the participants is equivalent to an average distribution, most of the man were shown mate choice relevant stimuli and women not. In future studies, this could be addressed with an inclusion of half nude male depictions as well as an assessment of participant’s sexual orientation and psychosexual identity. In case of our study, however, differences between male and female observers provide insights into gender specific effects in the processing of personalized female bodies regardless of sexual orientation, based on the ecological validity of a prevailing same versus other sex imbalance of body images in the media.

## Conclusion

Our findings of framing effects and gender differences in processing half nude female body representations can be set in a broader context of identifying a person based on personal information like a name or a place of living. In this regard, women positively respond to a more personalized version of female bodies that people are exposed to in the visual worlds of media in many cultures, i.e., women are more in favor than men of a ‘de-objectification’ of those body representations. The obtained results may inform the broader societal debate on the misuse of the female body in mass media in that personalizing a female body might be used to counteract its objectification.

## Author Contributions

SS: analysis of data; drafting the work; will be responsible for revising it and final approval of the version to be published. KF: substantial contributions to the conception or design of the work and the interpretation of the results. KE: preparation of stimulus material; contributions to the conception or design, acquisition of data, interpretation and manuscript preparation. MP: data acquisition; contributions to conception and interpretation.

## Conflict of Interest Statement

The authors declare that the research was conducted in the absence of any commercial or financial relationships that could be construed as a potential conflict of interest.

## Footnotes

1 http://www.fil.ion.ucl.ac.uk/spm
